# Hypoxia induced amoeboid microglial cell activation in postnatal rat brain is mediated by ATP receptor P2X4

**DOI:** 10.1186/1471-2202-12-111

**Published:** 2011-11-04

**Authors:** Fan Li, Lei Wang, Ji-Wei Li, Min Gong, Liang He, Rui Feng, Zhen Dai, Shu-Qing Li

**Affiliations:** 1 Department of Pathophysiology, Faculty of Basic Medical Sciences, Kunming Medical College, Kunming, 1168 West Chunrong Road 650500, PR China; 2 Department of Physiology, Faculty of Basic Medical Sciences, Kunming Hai-Yuan Medical College, Kunming, 295 Haiyuan Road 650031, PR China; 3 The Fourth Affiliated Hospital of Kunming Medical College, Kunming, 176 Qingnian Road 650051, PR China

## Abstract

**Background:**

Activation of amoeboid microglial cells (AMC) and its related inflammatory response have been linked to the periventricular white matter damage after hypoxia in neonatal brain. Hypoxia increases free ATP in the brain and then induces various effects through ATP receptors. The present study explored the possible mechanism in ATP induced AMC activation in hypoxia.

**Results:**

We first examined the immunoexpression of P2X4, P2X7 and P2Y12 in the corpus callosum (CC) and subependyma associated with the lateral ventricles where both areas are rich in AMC. Among the three purinergic receptors, P2X4 was most intensely expressed. By double immunofluorescence, P2X4 was specifically localized in AMC (from P0 to P7) but the immunofluorescence in AMC was progressively diminished with advancing age (P14). It was further shown that P2X4 expression was noticeably enhanced in P0 day rats subjected to hypoxia and killed at 4, 24, 72 h and 7 d versus their matching controls by double labeling and western blotting analysis. P2X4 expression was most intense at 7 d whence the inflammatory response was drastic after hypoxia. We then studied the association of P2X4 with cytokine release in AMC after hypoxic exposure. In primary microglial cells exposed to hypoxia, IL-1β and TNF-α protein levels were up-regulated. Blockade of P2X4 receptor with 2', 3'-0-(2, 4, 6-Trinitrophenyl) adenosine 5'-triphosphate, a selective P2X1-7 blocker resulted in partial suppression of IL-1β (24% *vs *hypoxic group) and TNF-α expression (40% *vs *hypoxic group). However, pyridoxal phosphate-6-azo (benzene-2, 4-disulfonic acid) tetrasodium salt hydrate, a selective P2X1-3, 5-7 blocker did not exert any significant effect on the cytokine expression.

**Conclusions:**

It is concluded that P2X4 which is constitutively expressed by AMC in postnatal rats was enhanced in hypoxia. Hypoxia induced increase in IL-1β and TNF-α expression was reversed by 2', 3'-0-(2, 4, 6-Trinitrophenyl) adenosine 5'-triphosphate suggesting that P2X4 mediates ATP induced AMC activation and its production of proinflammatory cytokines.

## Background

Perinatal brain injury occurs in approximately 4 per 1000 births [1] with exposure to hypoxic-ischemic insults and premature delivery (before 37 weeks of gestation) as major contributing factors. The defining feature of neuropathology in hypoxia and ischemia encephalopathy is the injury susceptibility of the periventricular white matter (PWM) [2]. It has been reported recently that the preponderant amoeboid microglial cells (AMC) and their activation is associated with the periventricular white matter damage (PWMD) in neonatal brain after hypoxia [3,4]. AMC activation in hypoxia is followed by inflammatory response affecting the axons and oligodendrocytes [4], but the underlying mechanism leading to AMC activation has remained uncertain.

Burnstock [5] proposed the roles of nucleotides as neurotransmitters. Thus, during inflammation or cellular damage affecting the neurons, the adenosine 5'-triphosphate (ATP) level in the extracellular spaces is described to be markedly increased [6,7]. It has been reported that ATP and its receptor were involved in cell-to-cell communication in physiological and pathophysiological conditions [8]. We have shown previously that ATP is involved in hypoxia induced microglial activation [9]. It has been reported that on exposure to ATP, microglia exhibited changes in its electrophysiological properties [10] and increased expression of cytokines in BV2 microglial cell line as well as the primary cultured microglial cells [9,11,12]. Microglial cells are known to express P2X receptors [13]. ATP would then induce various effects through its receptors [8,11,12]. As a group of membrane cation-selective receptor channels, P2X receptors interact with extracellular ATP [13]. It has been reported that ATP elicits discrete currents of P2X4R on microglia/macrophages [14]. In the latter, P2X4 receptors are involved in microglial activity in some pathological conditions. For example, the upregulation of P2X4 in microglia appears to play a crucial role in contributing to neuropathic pain which has a close relationship with the inflammation [15]. Suppression of P2X4 expression by 2', 3'-0-(2, 4, 6-Trinitrophenyl) adenosine 5'-triphosphate) (TNP-ATP) and pyridoxal phosphate-6-azo (benzene-2, 4-disulfonic acid) (PPADS) prevents tactile allodynia [16]. Guo [17] had reported that P2X4 expression in experimental autoimmune encephalomyelitis (EAE) was confined to the macrophage cells that infiltrated from the blood, furthermore, P2X4 expression reflects the inflammation response during EAE. Taken together, the above studies strongly suggest that P2X4 expression in microglial cells/macrophages may take part in the inflammatory response in injuries of the central nervous system.

The neuroprotective action exerted by TNP-ATP and PPADS together with the possible use of the purinergic antagonists in the pharmacological treatment of morphine induced migration of microglial [18] and oxygen/glucose deprivation [19-21] have been considered. Wixey and co-workers [22] have reported increased expression of ATP receptor P2X4 in microglial cells at 7 days after 3-day old postnatal rats were exposed to hypoxia-ischemia. However, the expression of ATP receptor and its changes after hypoxia in AMC in postnatal age, as well as the relationship of P2X4 and cytokine expression after hypoxia has remained to be explored.

It is suggested from the above studies that both ATP and ATP receptors may be jointly involved in microglial activation. The present study sought to explore the possible mechanism of ATP induced AMC activation after hypoxia in the periventricular white matter and the roles of ATP receptors especially P2X4 in regulating hypoxia induced cytokine expression in PWMD.

## Results

### P2X4, P2X7 and P2Y12 immunoexpression in AMC in postnatal rat brain

**Figure 1:**
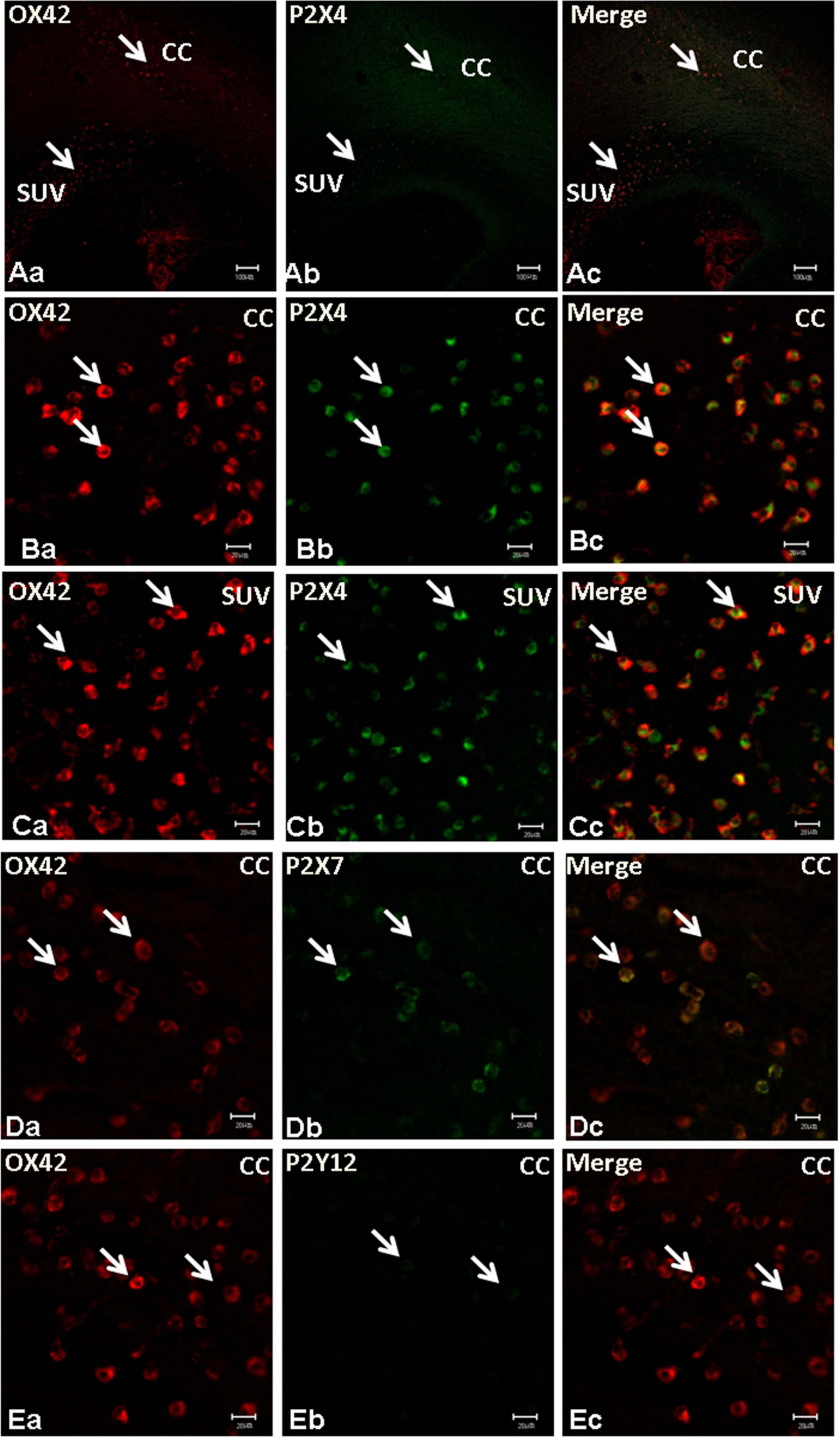
**P2X4, P2X7 and P2Y12 immunoexpression in AMC in postnatal rat brain**. By immunofluorescence labeling, P2X4 is colocalized with OX42 in the amoeboid microglial cells. Colocalization of OX42 and P2X4 in cells (arrows) is seen in the corpus callosum and subventricular zone (Aa-c, Scale bar = 100 μm). Immunopositive cells are round and have a typical morphology of amoeboid microglial cells (B/Ca-c. Scale bar = 20 μm). The amoeboid microglial cells exhibit a stronger P2X4 immunoreactivity compared with that of P2X7 and P2Y12 (D/Ea-c, Scale bar = 20 μm.).

By immunofluorescence double labeling, P2X4 AMC were distributed preferentially in the CC and subventricular zone (SVZ) in P3 rat brain (Figure 1Aa-c). There was a total coincidence between OX-42 and P2X4 in the AMC either in the CC (Figure 1Ba-c) or SVZ (Figure 1Ca-c). P2X7 and P2Y12 immunopositive cells were also found in the CC (Figure 1D-E) but the immunofluorescence intensity was noticeably less in comparison with P2X4 immunofluorescence. Furthermore, the frequency of AMC colabeled with P2X7 was lower compared to P2X4; even fewer AMC were positive for P2Y12. P2X7 and P2Y12-immunoreactive cells were confirmed to be the AMC as shown by double immunofluorescence labeling with OX42 (Figure 1D-E).

### P2X4 expression changes in AMC in P0 hypoxic rats

**Figure 2:**
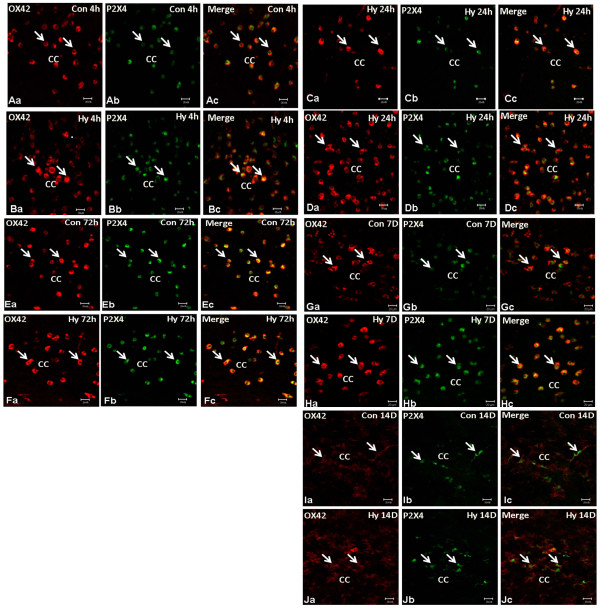
**P2X4 immunoexpression in P0 rats subjected to hypoxia (Hy) at different time points**. P0 rats subjected to Hy and sacrificed at 4 (Hy4 h), 24 (Hy24 h), 72 (Hy72 h), 7D (Hy7 d) and 14D (Hy14 d). Note P2X4 immunofluorescence in amoeboid microglial cells overlapping with OX42 fluorescence is noticeably enhanced in the hypoxic rats Hy4 h (A/Ba-c), Hy24 h (C/Da-c), Hy72 h (E/Fa-c), and Hy7 d (H/Ia-c) when compared with the matching controls (Con4 h, Con24 h, Con72 h, Con7 d and Con14d). Scale bar = 20 μm.

In P0 rats subjected to hypoxic exposure for 3 h and sacrificed at 4, 24, 72 h and 7, 14d, double immunofluorescence labeling showed the expression changes of P2X4 expression in AMC with advancing time. The fluorescence intensity and frequency of P2X4 positive cells were noticeably enhanced when compared with the respective matching controls at 4 h (Figure 2A-B), 24 h (Figure 2C-D), 72 h (Figure 2E-F), 7 d (Figure 2G-H), and 14 d (Figure 2I-J).

**Figure 3:**
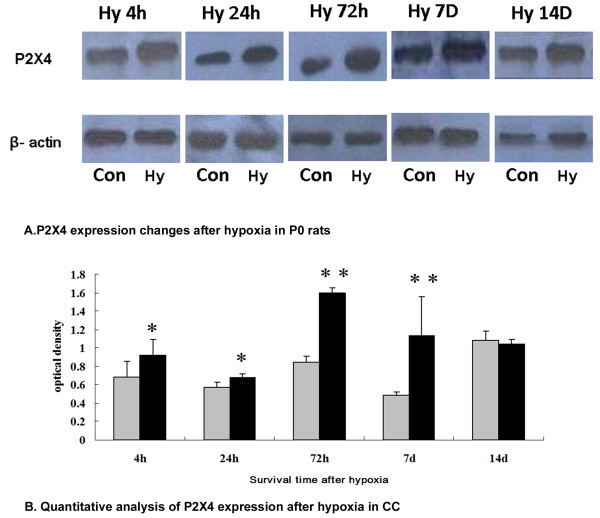
**P2X4 expression by western blotting in P0 rats subjected to hypoxia (Hy) at different time points in corpus callosum (CC)**. A. P2X4 expression after hypoxia in P0 rats. P0 rats exposed to Hy and sacrificed at 4 (Hy4 h), 24 (Hy24 h), 72 (Hy72 h), 7D (Hy7 d) and 14D (Hy14 d). It shows P2X4 (60 kDa) and β-actin (42 kDa) immunoreactive bands, respectively. P2X4 expression in CC after hypoxia is noticeably enhanced in the hypoxic rats at Hy4 h, Hy24 h, Hy72 h and Hy7 d when compared with the matching controls. B. Quantitative analysis of P2X4 expression after hypoxia in CC. P2X4 protein express in the CC at 3, 24, 72h, 7 and 14 d after the hypoxic exposure in P0 rats. In bar graph, relative P2X4 protein levels were normalized to β-actin levels in control and hypoxia groups. Data are mean ± SD, n = 6. *P < 0. 05 or **P < 0. 01 hypoxia *vs*. with their corresponding controls.

Western blot analysis showed that P2X4 protein expression level of the callosal tissue rich in AMC was up-regulated at 4, 24, 72 h and 7 d after hypoxia when compared with the matching controls (Figure 3). At 14 d after hypoxia, P2X4 expression level was comparable to that of the matching controls (Figure 3).

### TNF-α, IL-1β and P2X4 expression after hypoxia and blockade with PPADS/TNP-ATP in primary microglial cells

**Figure 4:**
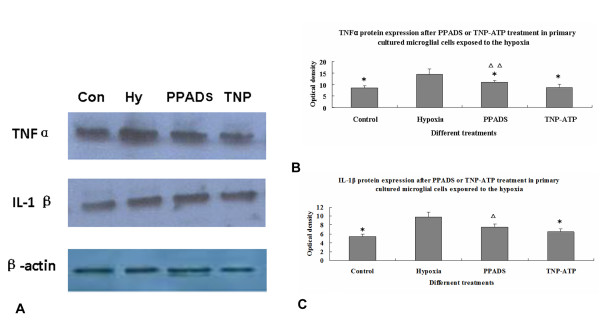
**Quantitative analysis of TNF-α and IL-1β protein expression in the primary cultured AMC after the hypoxic exposure coupled with PPADS or TNP-ATP treatment**. **A**. Western blotting of TNF-α at 30 kDa, IL-1β at 17 kDa immunoreactive bands, respectively. **B-C**. Bar graphs showing significant changes in the optical density of TNF-α and IL-1β, respectively, following hypoxic exposure when compared with their corresponding controls (mean ± SD). *P < 0. 01 Control, PPADS, TNP-ATP groups *vs*. hypoxic group, ^Δ^*P *< 0. 05 or ^ΔΔ^*P *< 0. 01 *vs*. TNP-ATP group. By western blotting analysis, IL-1β and TNF-α protein expression was significantly increased following hypoxia treatment. Blockade with PPADS, a blocker of P2X1-3, 5-7 resulted in decreased IL-1β and TNF-α protein expression after hypoxic exposure. A more drastic decrease in TNF-α and IL-1β protein expression was observed in hypoxic microglial cells blocked with TNP-ATP.

Following hypoxia, TNF-α and IL-1β mRNA expression in microglial cells *in vitro *was significantly increased. Additionally, by western blotting analysis, IL-1β and TNF-α protein expression was significantly increased following hypoxia treatment [9]. We next investigated the link between the IL-1β, TNF-α protein expression and P2X4 in hypoxic microglia. Primary microglial cells were blocked with PPADS, a blocker of P2X1-3, 5-7. This resulted in decreased IL-1β and TNF-α protein expression after hypoxic exposure. A more drastic decrease in TNF-α (Figure 4) and IL-1β (Figure 4) protein expression was observed in hypoxic microglial cells blocked with TNP-ATP.

### Morphological changes of AMC after hypoxia and following treatment with PPADS/TNP-ATP

**Figure 5:**
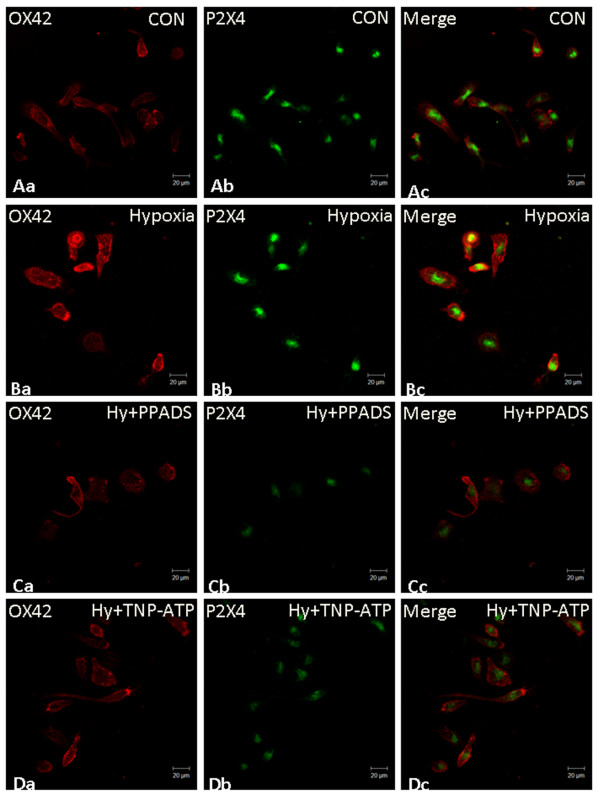
**P2X4 expression and phenotype change in primary cultured amoeboid microglial cells after the hypoxic exposure with PPADS or TNP-ATP treatment**. By immunofluorescence labeling, P2X4 is colocalized with OX42 in the primary cultured microglial cells (Aa-c, Scale bar = 20 μm). Following hypoxia, the phenotype of primary cultured AMC undergoes alteration (Ba-c, Scale bar = 20 μm) in which the cell body becomes rounded; however, the cells show long extending processes after treatment with PPADS (Ca-c, Scale bar = 20 μm). A more obvious change was found in hypoxic microglial cells treated with TNP-ATP (Da-c, Scale bar = 20 μm).

Following hypoxia, the external morphology of primary culture AMC underwent changes. The cell body of hypoxic microglia appeared to be more rounded. After PPADS treatment, the cells emitted long extending processes. Indeed, the cells appeared more ramified in TNP-ATP treated hypoxic microglial cells (Figure 5).

## Discussion

Microglial cells are motile immune-competent cells which assume a highly branched morphology in mature stage referred to as ramified microglial cells. In the early brain development, the microglial cells show a different phenotype characterized by a round or oval cell body with a variable number of stout processes and are referred to as the AMC [23]. They are preferentially located in the subcortical white matter notably the CC and the SVZ. The cells monitor the brain parenchyma under physiological conditions at different stages.

Recently, it has been reported [4] that AMC activation is involved in PWMD after hypoxia in the brain through the secretion of proinflammatory cytokines by the cell type. In this study, we have focused on the mechanism of AMC activation after hypoxia. It was hypothesized that AMC may be endowed a specific mechanism in their activation in hypoxia. In this connection, we have reported previously that AMC express voltage gated potassium channel Kv1. 2 which may be linked to hypoxia and lipopolysaccharide induced AMC activation. Additionally, ATP can induce Kv1. 2 expression and cytokine release in AMC after hypoxia [9]. It is well documented that free ATP is increased in hypoxic brain tissue [24,25]. It was surmised that ATP may be an important stimulus for microglial activation that may exacerbate the brain damage. This takes into consideration of the recent evidence that AMC are directly involved in PWMD causing damage to both the axons and myelin forming cells, oligodendrocytes [4,26]. Here, we provide evidence of the mechanism of hypoxia induced AMC activation after hypoxia, which is via ATP receptors.

### P2X4 expression during development

There is strong evidence indicating that neuronal degeneration in the brain typically develops as a result of microglial activation which is critically dependent on ATP receptor. ATP receptors are divided into ionotropic purinoceptor and metabolic purinoceptor. Ionotropic purinoceptors include P2X4 and P2X7, while metabolic purinoceptors include P2Y12. Ramified microglial cells express both ionotropic and metabolic purinoceptors [27]. However, expression of ATP receptors in AMC especially during development has remained to be explored. Indeed, there is only a modicum of information related to this by Wixey et al [22], who reported expression of P2X4 expression in microglial cells in postnatal rats at 10 days of age. More evidence for this would be desirable to confirm P2X4 localization in the AMC with typical morphology during development. We have confirmed this by double immunofluorescence labeling with OX-42 which demonstrated that P2X4 was indeed localized in AMC, but also extended the fact that P2X7 and P2Y12 were also localized in the same cell type but the expression was evidently less intense. It is striking that P2X4 is the predominant ATP receptor in the AMC in the developing brain while P2X7 and P2Y12 are detected in only a subpopulation of AMC. This differs from the ramified microglial cells in the adult rat brain [27]. In adult rat brain, P2X4 has been reported to be involved in some pathological conditions [28-30]. It is speculated that P2X4 may be actively involved in the AMC activation in early brain development whose function may be related to the susceptibility in injury of the PWM in hypoxia.

### Expression of P2X4 in AMC after hypoxia in postnatal rats

It has been reported that in some pathological conditions, expression of P2X4-receptor (P2X4R) a subtype of ionotropic purinoceptor increases in microglia, but not in neurons or astrocytes [28-30]. Additionally, P2X4 appears to play a crucial role in contributing to neuropathic pain [15,28,30] and P2X4 expression reflects the inflammation response during EAE [17]. Up-regulation of P2X4 expression was also detected in microglial cells at 7 days in a 3-day old rat's hypoxia-ischemia model [22], but the expression changes of P2X4 with time after hypoxia in P0 rats in AMC have remained to be fully clarified.

The present results have shown that increased P2X4 expression in AMC was sustained from 4 h to 7 d after hypoxia in newborn rats. This is evidenced by double immunofluorescence labeling of P2X4 in AMC at different time points. This is consistent with the western blot analysis which shows a significant increase in P2X4 expression in the callosal tissue when compared with the matching controls. It is to be noted that there is a significant increase in P2X4 expression at 72 h and 7 d after hypoxia. Wixey et al [22] reported increased P2X4 expression at 7 d only after hypoxia-ischemia injury. Here we showed that P2X4 expression was increased at an early time point after hypoxia in P0 rats. The discrepancy may be due to different experimental animal models used. In our study, we have used P0 rats subjected to hypoxic exposure. A marked increase in P2X4 expression was detected at P7 after hypoxia. Interestingly, this is the time point whence severe inflammatory response was noted in the PWM in a perinatal rats' hypoxic model [26].

At later time points e. g. at 14 d after hypoxia, the P2X4 expression was comparable to that in the matching normal controls. This suggests that while P2X4 expression in AMC is acute in onset in response to hypoxia, it subsides with time. A possible explanation for this may be that at early stage of hypoxia neuronal damage is more severe and, hence, extracellular ATP level is high. The concentration of free ATP in the brain may decrease at the later stage of hypoxia when cellular repair and regeneration are in progress. We have reported previously that a higher ATP concentration would elicit BV-2 microglial cell activation more profoundly [9]. A sudden surge in free ATP is expected in the acute and early phase of hypoxia. This would induce a stronger P2X4 expression at the early stage than the later stage of hypoxia. It has been reported that ramified microglia recruitment to the site of ischemia and hypoxia impairment is associated with cellular loss and P2X4-R are involved in cellular activation [19-21]. In this regard, the neuroprotective actions exerted by TNP-ATP and PPADS and the possible use of purinergic antagonist in the pharmacological treatment of oxygen/glucose deprivation have been considered [19-21,31]. Although P2X4 expression and inflammation after hypoxia-ischemia in the rat brain have been investigated after intraperitoneally injection of minocycline [22], the use of selected P2X4 antagonists to suppress AMC activation and associated inflammation in the postnatal brain after hypoxia has not been explored.

### P2X4 induced AMC activation and release of cytokines after hypoxia

There is ample evidence to suggest that ATP is involved in microglial activation specifically in the ramified phenotype in the adult brain [24,25,31]. On the other hand, the role of ATP in AMC activation especially the mechanisms in hypoxia have remained elusive. This is an important issue since AMC activation can lead to PWMD in perinatal hypoxia. During hypoxia damage affecting the neurons, the ATP level in the extracellular spaces is described to be markedly increased [24,25]. Here we detected an obvious increase in P2X4 expression in 7 d rats after hypoxia at P0, when the inflammatory response is most severe in this model [26]. Hence, we hypothesize that P2X4 mediates the inflammatory response in AMC subjected to hypoxia.

In the present study, primary microglial cells were subjected to hypoxia. Our results showed that after hypoxic exposure, IL-1β and TNFα protein expression was increased in primary microglial cells suggesting the activation of the cells. Along with this, the external microglial morphology also was altered. Additionally, increased P2X4 expression in primary microglial cell after hypoxia was also detected by confocal microscopy. We reported previously microglial activation after ATP exposure. Both IL-1β and TNF-α mRNA and protein levels were significantly increased after ATP treatment [9]. It was therefore concluded that ATP can induce microglial activation after hypoxia. In the present study, we confirmed that expression of ATP receptor P2X4 both *in vivo *and in primary cultured AMC.

Hypoxia induced AMC activation as reflected by increased production of TNF-α and IL-1β was suppressed following the application of PPADS and TNP-ATP, the selective blocker of P2X1-3, 5-7 and P2X1-7 [16], respectively. It is noteworthy that the external morphology of AMC returned to its resting state after treatment with both blockers suggesting that the latter can reduce activation of microglial cells. It is further suggested that P2X4 can regulate hypoxia induced AMC activation and take part in increased production of IL-1β and TNFα after hypoxia. Targeting P2X4 with its specific blocker may therefore be a potential therapeutic strategy to attenuate neuroinflammation in hypoxic brain in which high levels of ATP are expected to inundate the brain tissues.

The expression of TNFα can be blocked partially by PPADS; however, a more obvious suppression was exerted by TNP-ATP. Expression of IL-1β was suppressed only by TNP-ATP indicating its closer relationship to P2X4 expression in hypoxic microglia. The precise modulating mechanism of IL-1β and TNF-α expression after hypoxia awaits further investigation.

## Conclusion

We show here that AMC express ATP receptors P2X4, P2X7 and P2Y12 notably the first named receptor in hypoxia in neonatal brain. It is suggested that the prevalent ATP receptor P2X4 may be linked to regulation of AMC activation for production of proinflammatory cytokines especially in altered environment such as hypoxia. Therefore, unraveling the purinergic receptors, specifically P2X4 in its modulatory mechanisms for AMC activation may provide new therapeutic strategies for hypoxia induced injuries in the postnatal brain.

## Methods

The experimental procedures used in this study were approved by the Kunming Medical College Institutional Animal Care and Use Committee. In the handling and care of all animals, the International Guiding Principles for Animals Research, as stipulated by the Council for International Organizations of Medical Sciences (1985) and as adopted by the Laboratory Animal Centre, Kunming Medical College, Yunnan, China, were followed. All efforts were made to minimize the number of rats used and their suffering.

### P2X4 immunoexpression in amoeboid microglial cells in postnatal rats

Sprague Dawley (SD) rats at postnatal (P) 3 days (d) (P3) (n = 6) were used. The rats were anesthetized with 3% sodium pentobarbital (100 mg/kg) injected intraperitoneally and sacrificed by perfusion transcardially first with Ringer's solution, followed by fixation with 2% paraformaldehyde in 0. 1 M phosphate buffer. The brain was removed, post-fixed for 3 h in the same fixative, and then cryoprotected in 15% sucrose for 24 h. Frozen sections at 30 μm thickness were cut coronally through the forebrain with a cryostat (Leica model CM3050, Germany) and mounted onto gelatin-coated slides and stored at -20°C until use.

For ATP receptor immunofluorescence and double labeling with mouse anti-OX42 (CD11B) monoclonal antibody (1: 200, Milipore, Cat: CBL1512, USA), frozen coronal sections of the brain containing the CC rich in AMC were incubated with 10% horse serum (Vector, Cat: S-2000, USA.) for 40 min and rinsed in phosphate buffered saline (PBS) to block nonspecific protein. The sections were then incubated at 22-24°C with rabbit anti-P2X4 polyclonal primary antibody (1: 200, Alomone, Cat: APR-002, Yiselie), rabbit anti-P2X7 polyclonal primary antibody (1: 200, Alomone, Cat: APR-004, Yiselie), and rabbit anti-P2Y12 polyclonal primary antibody (1: 200, Alomone, Cat: APR-012, Yiselie) overnight. Subsequent antibody detection was carried out with FITC-conjugated donkey anti-rabbit IgG (1: 200, Millipore, Cat: AP182F, USA) for 1 h. After washing with PBS, the sections were incubated with Cy3-conjugated goat anti-mouse IgG (1: 200, Millipore, Cat: AP124C, USA) for OX42, a marker for microglial cells [32] for 1 h. The sections were then washed in PBS and mounted in a fluorescent mounting medium (DAKO Cytomation, Denmark). Cellular colocolization was then examined in a confocal microscope (Zeiss, LSM510 Axiovert 200 M, Germany).

### Hypoxic exposure of postnatal rats

P0 rats (n = 60) were kept in a hypoxia chamber (Chinese Utility Model Patent; patent number: ZL2010 2 01877. 4) filled with a gas mixture of 5% oxygen and 95% nitrogen for 3. 5 h. The rats were allowed to recover under normoxic conditions and killed at 4, 24, 72 h, 7, and 14 d (n = 6 at each time point for immunofluorescence or western blotting analysis) after hypoxia. For normoxic matching control, the rats (n = 6 for each time point for immunofluorescence or western blotting analysis) were kept outside of the chamber. For immunofluorescence study, the rats were sacrificed and the tissues processed as described above. Coronal brain sections were processed for P2X4 immunohistochemistry and immunofluorescence double labeling. For western blotting, the tissue was removed and stored at -80°C until use. The protein was extracted using a protein extraction kit according to the manufacturer's protocol. Western blotting was used to detect the expression levels of P2X4.

### Primary microglial cell culture

Highly purified microglial cultures were prepared from a modified method [33,34] using P2-P3 SD pups. The meninges were carefully removed from the brain and the cortical tissue was minced with a sterile scalpel blade and digested with 0. 25% trypsin/EDTA (HyClone, SH30042. 01, USA) for 15 min at 37°C. Five milliliters of Dulbecco's modified Eagle's medium high glucose (DMEM) (HyClone, Cat: SH30243. 01B, USA) supplemented with 10% charcoal-stripped fetal bovine serum (HyClone, Cat: SV30087. 02, USA) and 1% penicillin/streptomycin (HyClone, Cat: SV30010, USA)(P/S, 100 U/mL penicillin, 100 lg/mL streptomycin, P/S) (DMEM complete) were added to the tissue on ice, and the tissue was triturated with a 5 ml pipette several times, allowing the tissue clumps to settle. The supernatant was removed to a clean 50 ml conical tube on ice between triturations. Triturations were repeated until no tissue clumps were observed. The final volume was diluted to 25 mL medium and centrifuged at 3000 rpm for 5 min. The supernatant was discarded and cells resuspended in medium. A small aliquot of cells was stained for trypan blue exclusion and cells were plated at 10 million cells per 75 cm^2 ^flask. Cultures were maintained at 37°C and 5% CO_2_. The medium was changed the first time after 24 h, and then twice a week. After 10 days the bottom of flask was confluent with astrocytes and microglia. The flasks containing a mixture of astrocytes and microglia were shaken by hand for 2 min. Microglial cells in the medium were plated into 75 cm^2 ^flask or cover slip for different use. After 15 min, the medium was changed and the cells adherent to the bottom will be kept in the incubator for 24 h until use. About 98% of cells in cultures were identified to be microglia by staining with OX42 antibody, a marker for the microglial CR3/CD11B receptor.

### IL-1β and TNF-α expression in primary culture microglial cells after hypoxia

Primary culture microglial cells were seeded in a 75 cm^2 ^TC-treated culture flasks. They were then kept in the hypoxia chamber containing a gas mixture of 3% oxygen, 5%CO_2 _and 92% nitrogen at 37°C for 4 h. The protein was extracted using a protein extraction kit according to the manufacturer's protocol. Western blotting was used to detect the expression levels of IL-1β and TNF-α.

### P2X4 receptor blocking in primary culture microglial cells

Primary culture microglial cells were prepared as described above. Both PAADS (Sigma, Cat: P178, USA) and TNP-ATP (Sigma, Cat: T4193, USA) were used for blockade of P2X4. PAADS block P2X1-3, 5-7 receptors, while TNP-ATP blocks P2X1-7 receptor selectively. By comparing the blockade efficiency of the two blockers, information on the role of P2X4 in regulation of AMC activation is obtained [18,35].

PPADS at 30 μM and TNP-ATP at 20 μM were added into the primary culture microglial cells, and incubated for 2 h at 37°C in the chamber before hypoxia. After the hypoxic exposure, the protein was collected from the microglial cells for western blot analysis.

### Western blotting

Briefly, proteins were extracted from primary cultured microglial cells or postnatal rat brain tissue using a protein extraction kit (Biovision, Cat: K269-500, USA) according to the manufacturer's protocol. Cerebral tissues containing the CC were rapidly removed and immediately placed in dry ice before being stored at -80°C until use. The brain tissue was gently homogenized in ice-cold PBS (pH 7. 4) and centrifuged at 12000 g for 10 min at 4°C. The supernatant was recovered and frozen at -80°C. Total protein was determined using a commercial BCA kit (Pierce, IL, USA). Samples of supernatant containing 25 μg of protein were heated to 95°C for 5 min and were separated by sodium dodecyl sulfate-polyacrylamide gel electrophoresis in 12% gels, in a Mini-Protein 3 apparatus (Bio-Rad Laboratories, Hercules, CA, USA). After separation, proteins were transferred to a polyvinylidene difluoride membrane (Minipore, Cat: IPVH00010, USA) at 0. 8 mA/cm^2 ^of membrane for 2. 5 h in mixture solution of Tris-Glycine buffer (TBS) and 20% (v/v) methanol. After transfer, the membranes were blocked with 5% non-fat dry milk in TBS-Tween 20 0. 1% (TBST) and incubated with rabbit anti-P2X4 (1: 400), rabbit anti-β-actin (1: 5000, Abcam, ab8227, USA), rabbit anti-TNF-α polyclonal IgG (1: 500, Chemicon, Cat: AB1837P, USA) and rabbit anti-IL-1β polyclonal IgG(1: 500, Chemicon, Cat: AB1832P, USA) overnight, at 4°C. The antibodies were detected using horseradish peroxidase conjugated anti-rabbit IgG (1: 2500, KPL, Cat: 14-13-06, USA) secondary antibody and visualized using a chemiluminescence substrate system kit from Super Signal West Pico (KPL, Cat: 54-61-00, USA) on X-ray film (Super RX FujiFilm, Hanimax, QLD, Australia). Precision pre-stained standards (Bio-Rad Laboratories, Cat: 161-0376, USA) were used as molecular weight markers. X-ray films were scanned to quantify the band optical density using an Image J software (National Institutes of Health, USA). Figures with the quantitative western were derived from 6 animals from each group and the hybridizations of each animal were repeated at least 3 times.

### Statistical analysis

A two tails Student's t-test was used to determine the statistical significance of difference in values between the control and experimental preparations. A value of *P *< 0. 05 or *P *< 0. 01 was considered significant.

## Authors' contributions

All authors read and approved the final manuscript.

This study was designed by LF, who overseen the study entirely and accomplished the double immunofluorescence staining. WL and LJW operated hypoxic animal model and extracted the protein from the rats' brain for western blotting. GM prepared the primary cultured and hypoxic microgial cells for in vitro study. HL performed the statistical analysis for our data. FR carried out the Western blotting partially. DZ helped to draft the manuscript. LSQ was responsible for vetting, editing and approving of the manuscript.
